# Role of Wnt/*β*-Catenin Signaling in the Chemoresistance Modulation of Colorectal Cancer

**DOI:** 10.1155/2020/9390878

**Published:** 2020-03-18

**Authors:** Shengli Yuan, Fengying Tao, Xinglin Zhang, Yan Zhang, Xingxing Sun, Dapeng Wu

**Affiliations:** ^1^Department of Oncology, Qingdao Municipal Hospital, Qingdao 266011, China; ^2^Department of Oncology, Shanghai Jiao Tong University Affiliated Sixth People's Hospital, Shanghai 200233, China

## Abstract

Colorectal cancer (CRC) is a common malignancy with high morbidity and mortality worldwide. To date, chemotherapy plays an important role in the treatment of CRC patients. Multidrug resistance (MDR) is one of the major hurdles in chemotherapy for CRC, and the underlying mechanisms need to be explored. Studies have demonstrated that Wnt/*β*-catenin signaling plays a critical role in oncogenesis and tumor development, and its function in inhibiting apoptosis could facilitate tumor chemoresistance. Recent investigations have also suggested the regulatory effects of the Wnt/*β*-catenin signaling pathway in response to chemotherapeutic agents in CRC. Here, we particularly focus on reviewing the evidences suggesting the mechanisms of Wnt/*β*-catenin signaling in the chemoresistance modulation of colorectal cancer.

## 1. Introduction

Colorectal cancer (CRC), one of the most prevalent malignancies, ranked third in cancer incidence in both genders and remains the second cause of cancer-related deaths in the world [1]. Currently, chemotherapy and surgery are two major therapeutic approaches for CRC. Despite remarkable improvements in therapeutic strategies, the 5-year survival rate of CRC remains poor. For metastatic or advanced tumors, surgical resection alone fails to be curative. Systemic chemotherapy, aiming to prolong life and palliate symptoms, could shrink the tumor size before surgery and reduce recurrence after surgery. Progresses in combination chemotherapy such as FOLFOX, XELOX/CAPOX, FOLFIRI, and therapeutic antibodies against vascular endothelial growth factor (VEGF) and epidermal growth factor receptor (EGFR) have been shown to increase survival time. However, the emergence of multidrug resistance (MDR), accounting for the poor tumor response to antineoplastic agents, has greatly limited the efficacy of chemotherapeutic drugs and finally results in therapy failure in CRC patients [2–4]. Due to primary or acquired resistance, many patients either respond poorly to the chemotherapy or respond well initially but experience later tumor relapse and disease progression. Thus, it is urgent to understand the mechanisms responsible for MDR in CRC and develop effective strategies to overcome chemoresistance.

## 2. Mechanisms of Chemoresistance in Colorectal Cancer

Multidrug resistance is a major impediment to successful treatment of CRC, and overcoming MDR becomes a great challenge in fighting against CRC [5]. Chemotherapeutic drugs work through multiple mechanisms, often by targeting fast proliferating cells and inhibiting physiological DNA processes. MDR in CRC appears to be mediated by a series of mechanisms which could be divided into two principal types of resistance: “pump” and “nonpump.” The main mechanism of pump resistance is the increased ability of tumor cells to efflux drugs, which is induced by the ATP-binding cassette (ABC) superfamily of membrane transporters, including pump P-glycoprotein (P-gp), breast cancer resistance-associated protein (BCRP), and multidrug resistance-associated protein (MRP) subfamily. These transporters could pump out chemotherapeutic agents and reduce the accumulation of intracellular drugs, leading to an impairment of chemotherapeutic effects [5, 6]. The “nonpump” resistance mechanism includes apoptosis inhibition by Bcl-2 or p53, detoxification by GSTP1, cancer stemness cell- (CSC-) mediated multidrug resistance, epithelial–mesenchymal transition (EMT), altered tumor microenvironment, and functions of some noncoding RNAs. If CRC cells could negate the effects of chemotherapeutic agents by inhibiting apoptosis or promoting their own growth through the above mechanisms, then, resistance is achieved.

## 3. Wnt/*β*-Catenin Signaling and Chemoresistance in Colorectal Cancer

**Figure 1:**
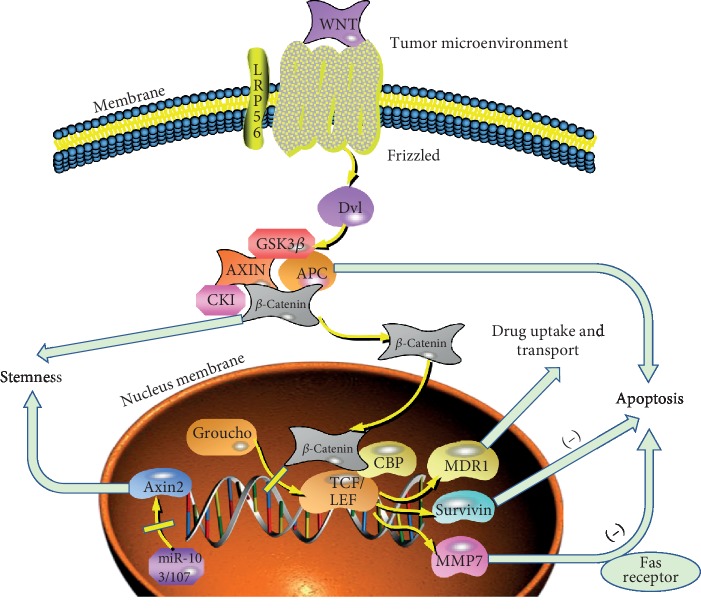
Role of the Wnt/*β*-catenin signaling pathway in chemoresistance of colorectal cancer.

The most common genetic changes accompanying CRC progression are mutations that deregulate the Wnt/*β*-catenin signaling cascade [7]. Wnt/*β*-catenin signaling pathway, essential for maintaining cell homeostasis and embryonic development, was demonstrated to be associated with tumor cell proliferation, apoptosis, invasion, stemness, and chemotherapy resistance [8] (Figure 1). Previous studies have identified the Wnt/*β*-catenin signaling pathway as a key pathway involved in various processes of CRC [9]. To date, many studies have demonstrated that loss of membranous *β*-catenin is associated with poor prognosis of CRC patients, and studies were performed to explore the roles and mechanisms of Wnt/*β*-catenin signaling in regulating cell apoptosis, stimulating angiogenesis, and maintaining highly resistant CSCs [10].

Wnt/*β*-catenin signaling pathway is found altered in more than 90% CRC patients, making it a crucial therapeutic target [11]. *β*-Catenin is a key regulator of the canonical Wnt/*β*-catenin signaling pathway, and elevated concentration of free *β*-catenin promotes the binding of the T-cell factor/lymphoid enhancer factor 1 (TCF/LEF) transcription factors, which control a cluster of target genes including MDR1, c-myc, Met, MMP-7, c-Jun, and cyclin D1. In this pathway, the cysteine-rich and lipid-modified Wnt proteins could induce the activation of canonical and noncanonical Wnt signaling pathways after binding to Frizzled (Frz) and low-density lipoprotein receptor-related 5/6 (LRP5/6). In physiological conditions lacking the Wnt signal, *β*-catenin is degraded by the complex composed of glycogen synthase kinase 3*β* (GSK3*β*), Axin, adenomatous polyposis coli (APC), and casein kinase 1 (CK1). As a key scaffolding protein of the destruction complex, Axin becomes degraded and stops facilitating the destruction of *β*-catenin after ribosylation by Poly (ADP ribose) polymerases (PARPs). Followed by proteasomal degradation and ubiquitination, *β*-catenin is phosphorylated by GSK3*β* and CK1. Downregulated cytoplasmic *β*-catenin recruits the corepressor Groucho to TCF/LEF, ensuring transcriptional repression and blocking the target genes from being activated. Given the tight association between Wnt/*β*-catenin signaling and cancer apoptosis, EMT, stemness, and tumor microenvironment, the Wnt/*β*-catenin signaling pathway is thought to be associated with cancer chemoresistance. We then mainly discuss the underlying mechanisms by which the Wnt/*β*-catenin signaling cascade influences the drug resistance of colorectal cancer in the following sections.

## 4. Wnt/*β*-Catenin Signaling and Drug Availability in Colorectal Cancer Cells

One of the most typical mechanisms of MDR is that ABC membrane transporters pump various chemotherapeutic agents out of cells to reduce intracellular drug accumulation and attenuate drug-induced cytotoxicity [11, 12]. Most of the ABC transporters contribute directly to chemoresistance, and attenuating their efflux activity could significantly reverse the resistance [13]. P-Glycoprotein (P-gp), referred to as MDR1 (multidrug resistance protein 1) or ABCB1 (ATP-binding cassette subfamily B, member 1), is the most crucial ABC transporter in human gastrointestinal system [14, 15]. In the Wnt/*β*-catenin signaling cascade, nuclear *β*-catenin could preferentially recruit CBP to the promoter region of MDR1 gene, and MDR1 has been proven to be one of the target genes of TCF/LEF [16, 17]. The MDR1 gene promoter was found to contain many T-cell factor 4- (TCF4-) binding sequences, and this gene was demonstrated to be transcriptionally downregulated after TCF4 inactivation in CRC, suggesting MDR1 is a direct target of the TCF4/*β*-catenin transcriptional complex [18]. Depletion of endogenous *β*-catenin by RNA interference could significantly reduce the transcription and expression of the MDR1 gene, resulting in a reversal of its encoded P-gp efflux and restoration of sensitivity to drug-induced apoptosis. Zhou et al. [19] showed that miR-506 could enhance the sensitivity of CRC cells to oxaliplatin via inhibition of the Wnt/*β*-catenin signaling pathway by suppressing MDR1/P-gp expression. Moreover, RARg overexpression was shown to contribute to the multidrug resistance of CRC cells by upregulating MDR1 through activating the Wnt/*β*-catenin signaling pathway [20]. Wang et al. [21] also demonstrated that suppressing TrpC5 expression could reverse 5-FU resistance in colorectal cancer by weakening the ABCB1 efflux pump through inhibiting the canonical Wnt/*β*-catenin signaling pathway. The above studies suggested that the Wnt/*β*-catenin signaling cascade contributed to enhanced resistance of various chemotherapeutic agents in CRC through upregulating MDR1.

## 5. Wnt/*β*-Catenin Signaling and Cell Apoptosis in Drug Resistance of Colorectal Cancer

Apoptosis, one of the major signs of effective chemotherapy, is characterized by DNA fragmentation, condensation of the nucleus, and specific protein degradation. Apoptosis resistance, a hallmark of tumors, acts as a crucial obstacle to anticancer therapy. As a critical mode of cell death induced by chemotherapy, apoptosis participates in chemotherapy resistance and plays a crucial role in regulating tissue homeostasis. Tolerance to DNA damage could be enhanced in chemoresistant cells through elevating the toxicity threshold by upregulating prosurvival signaling and downregulating apoptotic signaling pathways. The Wnt/*β*-catenin signaling cascade is a prosurvival signaling pathway that has an intimate crosstalk with other prosurvival signaling pathways such as signal transducer and activator of transcription (STAT), mitogen-activated protein kinase (MAPK), and phosphoinositide 3-kinase (PI3K)/Akt signaling pathways.

Wnt/*β*-catenin signaling pathway has been increasingly established to be associated with apoptosis. Previous evidence showed that inhibition of Wnt/*β*-catenin signaling by the inhibitor XAV939 could significantly increase the apoptosis induced by 5-FU in colon cancer cells [22]. As the key molecular of Wnt/*β*-catenin signaling pathway, *β*-catenin could promote the expression of its target gene survivin, which inhibits apoptosis in colon cancer [23]. Other components of the Wnt/*β*-catenin signaling pathway, including proteins Wnt, GSK3*β*, and APC, also participate in the process of apoptosis in CRC. As a member of Wnt proteins initiating the canonical Wnt signaling pathway, Wnt1 inhibits apoptosis of colorectal cancer cells through blocking the caspase-9 activation induced by chemotherapeutic drugs, and this sensitivity to apoptotic stimuli could be blocked by inhibiting *β*-catenin/TCF transcription [24]. As a serine threonine kinase, GSK3*β* could constitutively phosphorylate *β*-catenin and serve as a negative regulator of the Wnt/*β*-catenin signaling pathway [25]. Dewi et al. [26] showed that inhibition of GSK3*β* could increase the apoptosis of CRC cells. Adenomatous polyposis coli (APC), another crucial component of the canonical Wnt signaling cascade, could downregulate *β*-catenin. As a tumor suppressor protein, APC could induce cell death of CRC through apoptosis in CRC [27]. Moreover, downstream target genes in the Wnt/*β*-catenin signaling cascade have been reported to modulate drug resistance through regulating apoptosis. For instance, MMP-7 could increase the oxaliplatin resistance of colon cancer cells by decreasing the Fas receptor that promotes cell apoptosis [28]. Lastly, apoptosis-related proteins showed significant roles in regulating chemoresistance. Yang et al. [29] reported that Vicenin-2 induces apoptosis in colon cancer by suppressing Bcl-2 and enhancing the expression of Bax and caspase-3 through Wnt/*β*-catenin signaling. Li et al. [30] demonstrated that silencing aquaporin-5 could enhance the sensitivity of CRC cells to 5-FU by inducing apoptosis through the Wnt/*β*-catenin signaling pathway. Similar results were also observed in Chinese medicine including luteolin and Sanguisorba officinalis which could induce apoptosis by enhancing the Bax and caspase-3 expression and suppressing Bcl-2 expression through Wnt/*β*-catenin signaling in CRC cell lines [31, 32]. MASTL induces resistance to 5-fluorouracil (5-FU) through regulating antiapoptotic proteins survivin and Bcl-xL via the Wnt/*β*-catenin signaling pathway [33]. Thus, the crucial roles of Wnt/*β*-catenin signaling in apoptosis give it a status in the chemoresistance of CRC, and the underlying mechanisms need further exploration.

## 6. Wnt/*β*-Catenin Signaling and Colorectal Cancer Stem Cells in Drug Resistance

Cancer stem cells (CSCs) are a small subpopulation of cells that are endowed with the ability to self-renew, and differentiate into heterogeneous cell lineages that constitute the tumor [34, 35]. CSCs participate in tumor initiation and progression, playing a critical role in tumor proliferation, relapse, and metastasis [36]. Since CSCs express MDR1 intrinsically and own advantages in enhanced DNA repair capacity as well as high antiapoptotic signaling activation, they are also thought to be closely related to tumor chemoresistance [37]. It has been reported that drug-resistant tumor cells display a stem-like signature [38]. Chemotherapeutic strategies that kill bulks of tumor cells may fail at last, partly because they fail to eliminate CSCs and then result in the relapse of tumors [39]. It has been proven that CSCs display resistance to chemotherapeutic drugs through overexpressing ATP-binding cassette- (ABC-) family transporters, which act as drug-efflux pumps [40]. In colorectal cancer, several stem cell markers including Bmi1, Nanog, and CD44 have been identified, proving the existence of CSCs in CRC [41, 42]. However, the underlying molecular mechanisms how CSCs contribute to the chemoresistance in colorectal cancer remain unclear.

It has been proven that Wnt/*β*-catenin signaling modulates the expression of CSC marker genes and plays a role in the self-renewal ability and undifferentiated status of CSCs [43–45]. Accumulating evidences suggest that the Wnt/*β*-catenin signaling pathway, which regulates normal stem cell differentiation and proliferation, is important in maintaining cancer stem cell properties [46, 47]. Urushibara et al. [48] demonstrated that the Wnt/*β*-catenin signaling inhibitor IC-2 reduced the expression levels of CSC marker proteins and increased the cytotoxicity of 5-FU in CRC cells. As a negative feedback regulator of Wnt/*β*-catenin signaling, Axin2 could control Wnt-induced transcriptional responses. Suppression of Axin2 by miR-103/107 was demonstrated to enhance CRC chemoresistance by promoting cell stemness via Wnt/*β*-catenin signaling [49]. Moreover, accumulation of nuclear *β*-catenin enhances both the chemoresistance and radioresistance of locally advanced rectal cancer through regulating EMT/CSC properties, and nuclear *β*-catenin in pretreatment-biopsied samples is promising in predicting the efficacy of chemoradiotherapy in rectal cancer patients [50]. Liu et al. [51] showed that CD146 decreased the drug resistance of colorectal cancer by functioning as a suppressor of cancer stemness through inactivating the Wnt/*β*-catenin cascade. It was also observed that zerumbone could suppress the stemness properties of CRC by inhibiting the *β*-catenin signaling pathway [52]. These studies all suggested that the Wnt/*β*-catenin signaling pathway plays crucial roles in fostering chemoresistance of CRC through stemness.

## 7. Wnt/*β*-Catenin Signaling and Colorectal Cancer Epithelial–Mesenchymal Transition in Drug Resistance

Recently, accumulating evidence suggests molecular and phenotypic associations between epithelial–mesenchymal transition (EMT) phenotype and tumor chemoresistance [53–55]. EMT is a process that allows epithelial cells to undergo remarkable morphologic changes to assume a migratory mesenchymal phenotype characterized by loss of apical basolateral polarity and cell-cell adhesion [56, 57]. When the process of EMT is triggered, epithelial cells interacting with the basement membrane through the basal surface downregulate the expression of adhesive proteins, such as E-cadherin and acquire the expression of mesenchymal markers, such as N-cadherin, MMP-2, MMP-9, Vimentin, and fibronectin. These alternations, which bring reorganization of the actin cytoskeleton and deficiency of cell–cell junction, are often usurped by tumors to enhance invasion, mobility, and proliferation [58]. Moreover, studies have proven that EMT is a crucial way to induce CSC formation in many tumors, and induction of EMT confers properties of self-renewing stem cells, suggesting a close relation between EMT and the acquisition of stem cell characteristics [36, 58, 59].

Since studies have reported that residual resistant cells following chemotherapy are associated with an EMT phenotype in animal models as well as in clinical settings, EMT has now emerged as the focus of research into the cause of chemoresistance in several tumor types [60–63]. The occurrence of EMT was proven to be closely associated with the activation of intracellular stem-associated pathways including Wnt/*β*-catenin, Notch, TGF-*β*, and Hedgehog pathways while the underlying mechanisms have not been clearly defined [64, 65]. As one of the major signaling pathways involved in EMT, Wnt/*β*-catenin signaling converging on activation of transcription factors such as ZEB and Snail was triggered to induce the expression of mesenchymal genes and repress E-cadherin expression [57, 66]. *β*-Catenin and E-cadherin form a complex in the area of cell–cell junction, providing the basis for cell–cell association [67]. Previous studies have shown that ectopic expression of Snail and E-cadherin causes EMT in colorectal cancer [68–70]. In CRC, loss of E-cadherin is concomitant with the deregulation of the Wnt/*β*-catenin signaling pathway and has been characterized as a trait of EMT cells [71]. Chen et al. [72] showed that nuclear translocation of membrane *β*-catenin and disassociation of the E-cadherin/*β*-catenin complex activate *β*-catenin–TCF transcription, decrease E-cadherin levels, and increase Snail expression. CRC metastasis could be also promoted by inducing EMT through a *β*-catenin–dependent pathway [72]. Another study by Qi et al. [73] demonstrated that Wnt3a overexpression leads to the distribution of cytosolic *β*-catenin, downregulation of epithelial markers, and overexpression of mesenchymal markers, in both cellular and animal models of colorectal cancer. Collectively, Wnt/*β*-catenin signaling may modulate the chemosensitivity of CRC through EMT.

## 8. Wnt/*β*-Catenin Signaling and Tumor Microenvironment in Drug Resistance of Colorectal Cancer

Although the investigation of chemoresistance in CRC has been focused on mechanisms intrinsic to tumor cells, alternative views propose a role for the tumor microenvironment (TME) in promoting chemoresistance. The tumor microenvironment, regarded as the tumor bed, contains components including extracellular matrix (ECM) proteins, aberrant vasculature, and cancer-associated cells [74]. TME also contains many paracrine factors and signaling molecules that initiate intracellular signaling within tumor cells and crosstalk between cancer cells and the surrounding supportive stromal cells.

In stromal cells, it is reported that hepatocyte growth factor (HGF) secreted by myofibroblasts could activate nuclear *β*-catenin activity and thereby affect stemness features which are associated with chemoresistance in colorectal cancer cells [75]. As the main component of the stroma, cancer-associated fibroblasts (CAFs), different from normal fibroblasts in the TME, exert inherent support on cancer cells via secretion of molecular messengers and cell-to-cell contact [74]. In colorectal cancer, CAFs could promote drug resistance by transferring exosomal H19, which activates the *β*-catenin signaling pathway via acting as a competing endogenous RNA sponge [76]. lncRNA CCAL (colorectal cancer-associated lncRNA) expressed by CAFs contributes to oxaliplatin resistance of CRC cells via activating *β*-catenin signaling pathway [77]. lncRNA CCAL was also reported to enhance multidrug resistance by upregulating MDR1/P-gp expression through activating the Wnt/*β*-catenin signaling cascade [18]. In immune cell populations, a study showed that tumor-induced *β*-catenin signaling infiltrates immune effector cells into a tolerant state and inhibits the DC-dependent cross-sensitization of antitumor CTLs [78]. Active forms of *β*-catenin promote resistance to immunotherapy with anti-PD-1, which impairs T-cell activity and involves the deficient recruitment of DCs [79]. As a critical feature in the tumor microenvironment, hypoxia self-perpetuates mainly through the regulation of the vasculature. Under hypoxia, the expression levels of miR-103 and miR-107 are elevated, and miR-103/107-Axin2 axis contributes to chemoresistance to oxaliplatin and cisplatin through prolonging Wnt/*β*-catenin signaling duration in HCT116 cells [49]. Hypoxia has also been shown to activate GLI2 via HIF-1*α* and TGF-*β*2 to promote chemoresistance in colorectal cancer. Nevertheless, the underlying mechanism was not related to the *β*-catenin signaling pathway [80, 81].

## 9. Wnt/*β*-Catenin Signaling and Noncoding RNAs in Drug Resistance of Colorectal Cancer

In recent years, an increasing number of studies have indicated that regulation of gene expression by various noncoding RNAs (ncRNAs) such as microRNAs (miRNAs) and long noncoding RNAs (lncRNAs) were involved in the acquisition of chemoresistance after treatment [82]. miRNAs are short, evolutionarily conserved noncoding RNAs that posttranscriptionally regulate gene expression by binding the 3′ untranslated regions (3′UTRs) of mRNAs. They have been increasingly implicated in the regulation of cell stemness and EMT, which contribute to drug resistance in colorectal cancer [83, 84]. Recent studies have shown that some miRNAs (e.g., miR-409-3p, miR-137, miR-139-5p, miR-494, and miR-143) reduced CRC chemoresistance, while some other miRNAs (e.g., miR-192, miR-587, miR-133a, miR-215, and miR-492) are associated with increased chemoresistance in CRC [85]. lncRNAs are mRNA-like transcripts lacking significant open reading frames and have been shown to regulate cell apoptosis, proliferation as well as drug functions in multiple tumor types [83]. Recent advance to date have strongly cemented the fact that lncRNAs could modulate the chemoresistance of CRC through miRNAs, and miRNAs could crosstalk with various key cellular signaling networks including the Wnt/*β*-catenin cascade [86, 87]. The association between ncRNAs and chemoresistance of CRC is increasingly established, and identification of specific ncRNAs may aid molecular targets for future relief of chemoresistant CRC. Thus, we listed the roles of ncRNAs in CRC chemoresistance through the Wnt/*β*-catenin signaling pathway in Table 1.

## 10. Conclusions and Perspectives

Chemoresistance remains a considerable challenge preventing better cure rates after treatment initiation in CRC nowadays. Unraveling the molecular mechanisms driving the chemoresistance of CRC would be beneficial for identifying invaluable therapeutic targets for clinical applications. As mentioned in the present review, chemoresistance related to *β*-catenin signaling in CRC has been shown to be associated with a variety of mechanisms including decreased intracellular drug accumulation, apoptosis inhibition, presence of highly resistant CSCs, EMT, tumor microenvironment, and some ncRNAs. The Wnt/*β*-catenin signaling pathway could be further investigated as a promising target in the development of new drugs to alleviate chemoresistance. Nowadays, preclinical studies and clinical trials showing patients' responses to therapy with inhibitors of Wnt/*β*-catenin signaling pathway are undergoing. Some experimental evidence from preclinical studies already suggests a beneficial consequence of *β*-catenin signaling pathway blockade. For example, as a soy-derived isoflavone, which could be used as an inhibitor of the Wnt pathway by inactivating *β*-catenin signaling through overexpressing GSK3*β* and E-cadherin, Genistein has been reported to play a role in reversing resistance to fluoropyrimidine compounds and platinum [93]. Moreover, whether some mechanisms that exist in CRC chemoresistance, such as epigenome, autophagy, and metabolism, are also associated with the Wnt/*β*-catenin signaling pathway need to be further explored in the future. Finally, more progresses in molecular biology enabling clinicians to reverse drug resistance of CRC are eagerly anticipated.

## Figures and Tables

**Table 1:** Summary of non-coding RNAs in modulating chemoresistance through the *β*-catenin signaling pathway in colorectal cancer.

Noncoding RNA	Sample	Drug	Cellular mechanism of action	Target	Ref.
miR-506	HCT116-OxR cells	Oxaliplatin	Increase oxaliplatin-induced cell apoptosis and enhance oxaliplatin sensitivity by inhibiting MDR1 expression via downregulation of the Wnt/*β*-catenin signaling pathway	MDR1	[19]
miR-103/107	HCT116 cell	Oxaliplatin, cisplatin	Promote CRC stem-like properties and prolong Wnt/*β*-catenin signaling duration by targeting Axin2	Axin2	[49]
miR-30-5p	CD133+CRC cells (Caco2 and HCT15)	5-FU	Inhibit CRC cell stemness and chemoresistance through USP22/Wnt/*β*-catenin signaling axis	USP22	[88]
miR-92a	HT-29 and HCT116 cells	5-FU	IL-6/STAT3/miR-92a/Wnt/*β*-catenin signaling pathway promotes stem-like phenotypes of colorectal cancer cells	KLF4, GSK3*β*, and DKK3	[89]
miR-100/125b	NCI-H508, Caco2, SW403, SW948, HT-29, SK-CO-1, etc.	Cetuximab	Repress multiple Wnt negative regulators and increase Wnt signaling	DKK1 and DKK3	[90]
lncRNA H19	HCT116 and SW480 cells	Oxaliplatin	Promote stemness and activate the *β*-catenin signaling pathway via acting as a competing endogenous RNA sponge for miR-141	miR-141	[76]
lncRNA CRNDE	HCT116 and SW480 cells	5-FU	lncRNA CRNDE promotes chemoresistance to 5-FU by inhibiting miR-181a-5p. MiR-181a-5p targets *β*-catenin/TCF4 and inhibits Wnt/*β*-catenin signaling.	miR-181a-5p	[91]
lncRNA CCAL	Lovo and Lovo/5-FU cells	5-FU	Induce multidrug resistance through activating Wnt/*β*-catenin signaling by suppressing AP-2*α* and further upregulating MDR1/P-gp expression	AP-2*α*	[18]
lncRNA HOTAIR	Colo205 and SW620 cells	Cisplatin, paclitaxel	Promote the chemoresistance of CRC cells through targeting miR-203a-3p-mediated Wnt/*β*-catenin signaling pathway	miR-203a-3p	[92]
